# The Effectiveness and Safety of Acupoint Catgut Embedding for the Treatment of Postmenopausal Osteoporosis: A Systematic Review and Meta-Analysis

**DOI:** 10.1155/2019/2673763

**Published:** 2019-08-14

**Authors:** Fan Huang, Yumin Xie, Siyi Zhao, Zitong Feng, Guizhen Chen, Yunxiang Xu

**Affiliations:** ^1^Clinical Medical College of Acupuncture Moxibustion and Rehabilitation, Guangzhou University of Chinese Medicine, Guangzhou, 510405, Guangdong, China; ^2^The Bao'an District TCM Hospital, The Affiliated Hospital of Guangzhou University of Chinese Medicine, Shenzhen, 518133, Guangdong, China

## Abstract

**Purpose:**

To evaluate the effectiveness and safety of acupoint catgut embedding therapy (ACET) in postmenopausal osteoporosis (PMOP).

**Methods:**

Review of some databases from their inception to June 2018 and randomized controlled trials (RCTs) in which ACET with PMOP were included. Two researchers extracted and evaluated the information independently. Cochrane Collaboration's tool and Jadad scale were used to evaluate the quality of the studies. RevMan V.5.3.3 software was used to carry out the meta-analysis while trial sequential analysis (TSA) performed with TSA 0.9 software.

**Results:**

12 RCTs with 876 participants were included in this review. Meta-analysis showed that ACET alone was not superior to medication in effectiveness rate (RR= 1.11; 95% CI (0.89, 1.40);* P*=0.35) and E2 (SMD= 0.20; 95% CI (-0.17, 0.57);* P=*0.28;* I*^*2*^=20%) while ACET combining medication was more effective on the effectiveness rate (RR= 1.32; 95% CI (1.20, 1.46);* P*<0.000 01) and E2 (SMD= 1.24; 95% CI (0.63, 1.84);* P*<0.0001). Additionally, ACET combining calcium could increase the bone mineral density (BMD) of the L2~4 vertebrae and femur-neck [WMD_L2~4_ = 0.03; 95% CI (0.01, 0.05);* P*=0.003; and WMD_Femur-neck_ = 0.07; 95% CI (0.03, 0.10);* P* = 0.0006], reduce TCM syndrome score [WMD = -1.85; 95% CI (-2.13, -1.57);* P*<0.000 01], improve patient's quality of life [WMD_three  months_ = 6.90; 95% CI (3.90, 9.89);* P*<0.000 01; and WMD_six  months_ = 12.34; 95% CI (5.09, 19.60);* P*=0.0009], and relieve pain [WMD_VAS_ = -1.26; 95% CI (-1.66, -0.85);* P*<0.000 01; and WMD_Pain  score_ = -2.59; 95% CI (-4.76, -0.43);* P*= 0.02]. The TSA showed that the effectiveness of ACET for PMOP was demonstrated accurately.

**Conclusions:**

ACET combining medication but not ACET alone is more effective than medication as comparison in the treatment of PMOP. As a novel treatment, ACET shows the potential of effectiveness and deserves further high quality of well-designed study.

## 1. Introduction

Postmenopausal osteoporosis (PMOP) is a common clinical disease characterized by bone mass reduction and microarchitecture degradation [1, 2] that contributes to an increased risk of bone fragility and fracture. PMOP is a major health threat, affecting 50% of postmenopausal women worldwide, and is considered one of the most common diseases among the elderly [3]. Approximately 200 million women have been diagnosed with osteoporosis worldwide [4], and 1.5 million fractures are caused by osteoporosis in USA [5]. The health care costs directly related to osteoporosis are currently estimated to range from 13.7 to 20.3 billion dollars in America [6]. PMOP is caused by a decrease in ovarian function and levels of oestrogen in postmenopausal women, which causes bone mass reduction, increases bone brittleness, and leads to an increased prevalence of osteoporosis [7]. High morbidity, a high incidence of pain, and high disability rates are hallmarks of the disease. These symptoms seriously affect the quality of life of many postmenopausal women [8]. There are currently many therapeutic options available for the disease; commonly used medications include alendronic acid, risedronic acid, vitamin D, and calcium, among others [9]. However, the long-term use of these drugs represents an economic burden for affected patients. Additionally, these medications have serious side effects in the gastrointestinal tract, even leading to endometrialgia, breast cancer, oesophageal cancer, kidney stones, and other issues [10].

There is no PMOP in Traditional Chinese Medicine (TCM); according to its aetiology and pathogenesis, it can instead be classified as osteanabrosis [11, 12]. Studies have shown that kidney deficiency is the pathological basis of PMOP. After menopause, bones lack necessary nourishment because of a deficiency in kidney functionality, which contributes to the atrophic debilitation of bones [13, 14]. Hence, the treatment of TCM should ideally be based on tonifying the kidney. Acupuncture is one of the characteristic therapies of TCM. Meta-analysis of acupuncture for the treatment of osteoporosis has indicated that warm acupuncture, electroacupuncture, and traditional acupuncture can improve clinical symptoms and provide pain relief in osteoporosis [15, 16]. However, there is currently no comprehensive review of ACET for the treatment of PMOP. Moreover, most of the clinical studies of ACET have utilized small sample sizes, and large-scale RCTs have not been carried out due to the long duration of treatments and subsequent visits, which may affect the measurement of the observed indicators. As a specialized form of acupuncture therapy, ACET can cause continuous needling effects at specific points through the insertion of catgut. This therapy is characterized by rapid short-term effects and sustained long-term effects [17]. Compared with other treatments, ACET has marked advantages in terms of overcoming the high cost of health care, reducing the toxicity and side effects of drugs, shortening the frequency and duration of traditional acupuncture treatments, and improving patient compliance [12, 18]. Additionally, compared with other acupuncture therapies, ACET has significant sustained stimulation effects; however, the positive effects of other clinical indicators remain to be confirmed. Therefore, this study systematically evaluated the effectiveness and safety of existing RCTs of ACET in the treatment of PMOP, to optimize acupoint interventions for the treatment of osteoporosis and provide a basis for clinical treatment.

## 2. Methods

We used the preferred reporting items for systematic reviews and meta-analysis (PRISMA) statement to guide our systematic evaluation report [19]. And the review was registered in the International Prospective Register of Systematic Reviews (PROSPERO) database on 17 January 2019 (registration no. CRD42019120548).

### 2.1. Study Searches

To collect RCTs of PMOP, two independent researchers searched the data bases: PubMed, EMbase, Cochrane, CNKI, VIP, CBM, and Wan Fang Data from their inception to June 18, 2018 (via their websites). The retrieval strategies combined subjects and free words, which were determined after multiple presearches. Whenever necessary, we contacted the original study authors to obtain additional information. Manual searches of relevant core journals, books, and references were also conducted to supplement the relevant studies. The search terms (“Postmenopausal Osteoporosis” OR “Postmenopausal Bone Loss”) AND (“Catgut Embedding Therapy” OR “Acupoint Catgut Embedding” OR “Catgut Implantation At Acupoint” OR “Point Embedding Therapy” OR “Acupoint Thread-embedding”) AND “Random” were used in the English-language databases, while* juejinghouguzhishusongzheng, xueweimaixian, *and* suiji *were used in the Chinese-language databases.

### 2.2. Inclusion Criteria

Studies involving RCTs in which subjects were diagnosed with PMOP and an experimental group was treated with ACET alone or in combination with other adjunct interventions, such as calcium supplements (e.g., calcium carbonate D3 chewable tablets or Leli capsules), were included. Trials must have reported at least one outcome indicator of improvement of osteoporosis, such as E2 levels or BMD, and the trial must have been published in English or Chinese.

### 2.3. Exclusion Criteria

Studies with the following characteristics were excluded: non-English or Chinese-language studies; studies without consistent diagnostic criteria or relevant outcome indicators; studies with other treatments used in the experimental group in addition to ACET; duplicate reports; studies with data that could not be extracted or merged because the samples or efficiencies were unspecified; studies enrolling participants with other diseases in addition to PMOP; case reports, comments, or review articles.

### 2.4. Data Extraction

After the search, two independent researchers (Fan Huang and Siyi Zhao) reviewed the retrieved articles to filter and extract information according to the inclusion and exclusion criteria. A third researcher (Zitong Feng) was consulted to resolve disagreements to ensure that all included studies were consistent. The extracted data included the study title, first author, year of publication, literature sources, baseline of patients in each group, intervention methods, outcomes, randomization methods, allocation and concealment methods, blinding methods, follow-up, and patient loss to follow-up or withdrawal, among other factors.

### 2.5. Quality Assessment

Methodological quality assessment was performed according to the Cochrane evaluation manual version 5.1.0 [20], and the Jadad scale was used to analyse the bias risk of the included RCTs [21]. The main items of the Cochrane evaluation manual version 5.1.0 include the specific randomized method, allocation concealment, the implementation of blinding methods, selective reporting, the integrity of the resulting data, the use of intentional therapy analysis (ITT analysis) if there were losses to follow-up or withdrawal, and other biases. According to the evaluation criteria of the Jadad scale, the included clinical trials were graded from 0 to 5 points. If the point value was ≤ 2, the trial was considered low quality; if the point value was > 2, it was considered high quality. The detailed scoring criteria were as follows: (1) application of a randomized method, (2) application of blinding method, and (3) application of withdrawal and loss to follow-up.

### 2.6. Statistical Analyses

RevMan V.5.3.3 software (Cochrane Collaboration, Oxford, UK) was used to carry out the meta-analysis, while the use frequency of various acupoints was analyzed with Microsoft Excel 2016. A trial sequential analysis was performed with TSA 0.9 software. Egger's test was used to assess publication bias with the Stata12.0. For dichotomous variables, relative risk (RR) estimates with 95% confidence intervals (CIs) were calculated. For continuous variables, weighted mean differences (WMDs) with 95% CIs were calculated. If* P* ≥ 0.01 and* I*^2^ ≤ 50%, the fixed effect model was adopted for the meta-analysis. Otherwise, the sources of heterogeneity were further analyzed. After excluding the influence of marked clinical heterogeneity, a random effects model was adopted to perform the meta-analysis. Sensitivity and bias risk analyses were also performed. Subsequently, the use frequency of acupoints was statistically analyzed using a column graph, and a trial sequential analysis of the total effectiveness rate was conducted [22]. Finally, we reported a general descriptive analysis of adverse reactions.

## 3. Results

### 3.1. Study Selection

**Figure 1:**
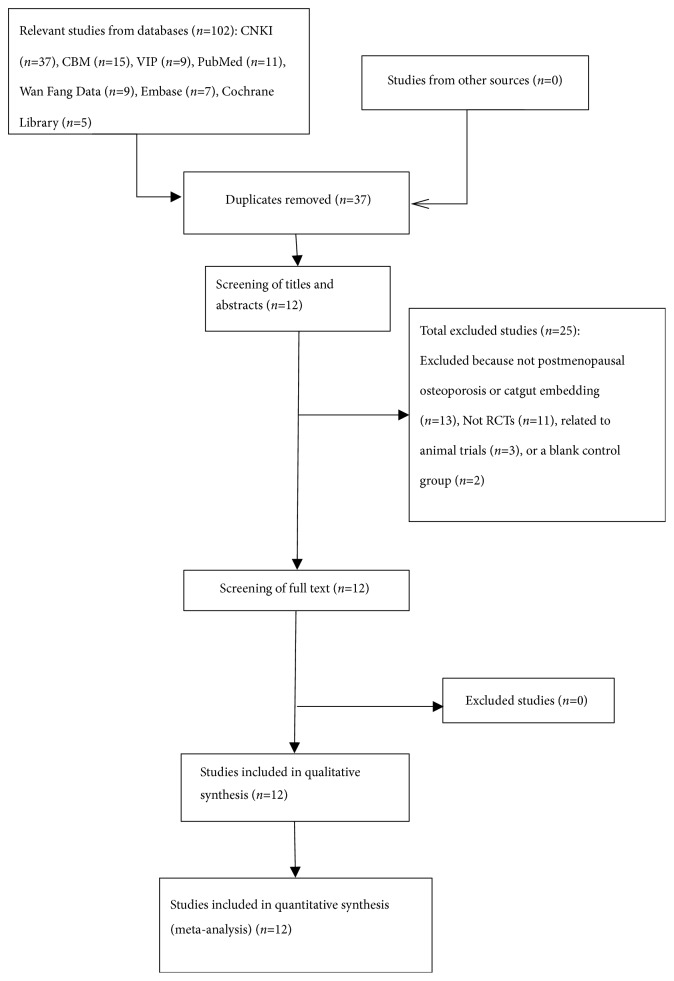
Flow diagram showing selection of the included studies.

We obtained 102 relevant studies through preliminary searches. After multiple filtering steps, 12 RCTs with a total of 876 participants were ultimately included in this systematic review [23–34].  Figure 1 is a flow diagram summarizing the selection of the included studies.

### 3.2. Characteristics of the Included Studies

Among the twelve included studies [23–34], five trials [23–27] compared ACET with medicine [24–27] or sham ACET [23]. In the remaining trials [28–34], ACET combining calcium supplements served as the experimental group, and calcium supplements alone served as the control group. The duration of intervention in these trials was either three months [23, 25–27] or six months [24, 28–34]. All the characteristics of the included studies are shown in Table 1.

### 3.3. Assessment of Bias Risk

**Figure 2:**
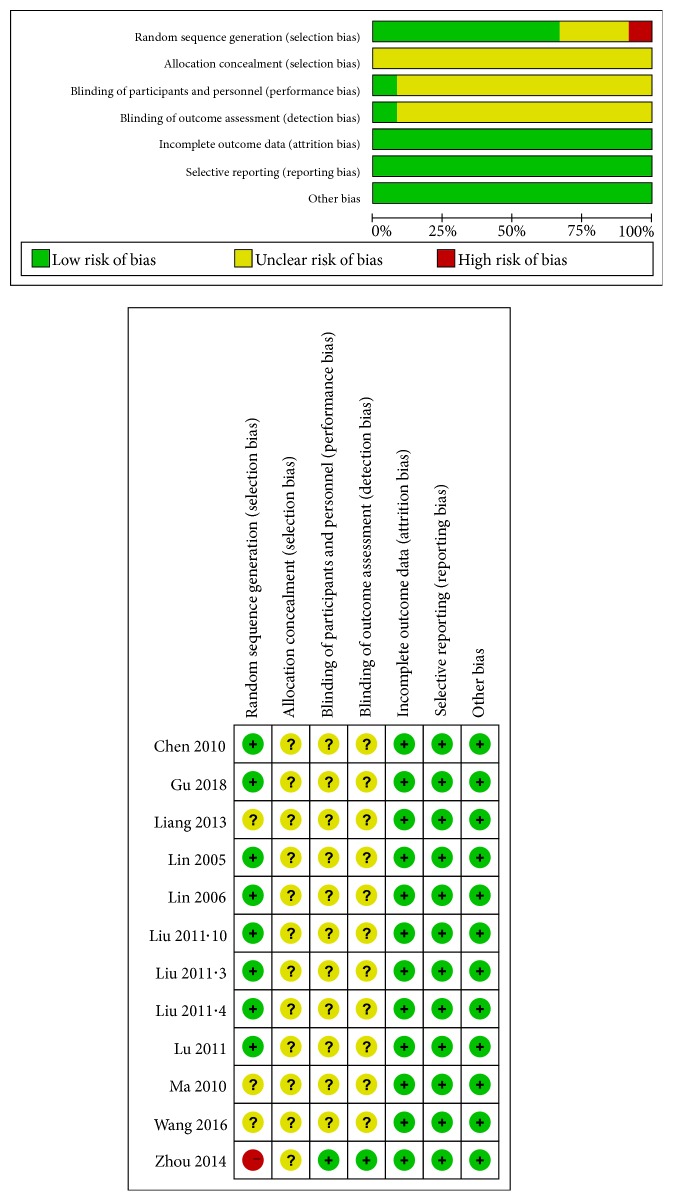
Bias risk assessment results of the included studies.

Bias risk assessment results of the included studies are shown in Figure 2.

### 3.4. Meta-Analysis

#### 3.4.1. Effectiveness Rate

**Figure 3:**
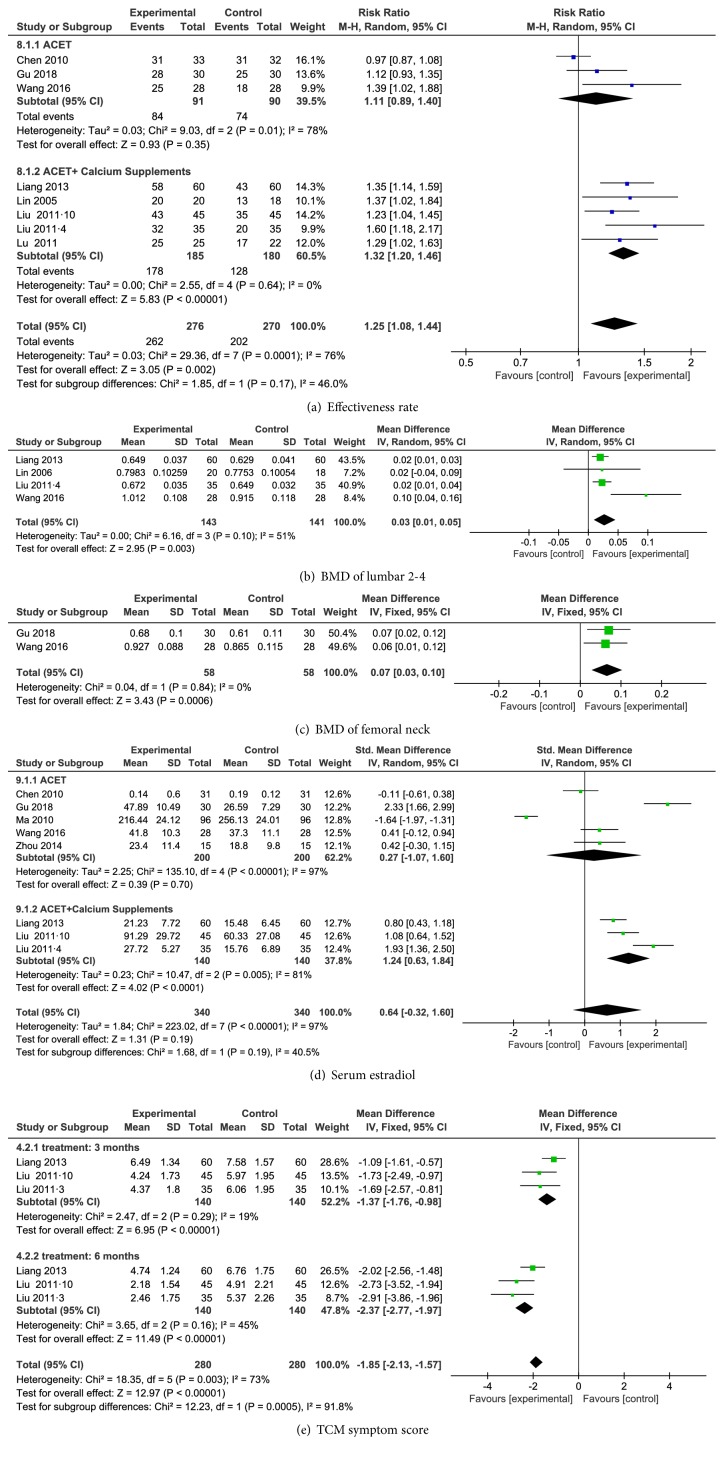
Forest figure of clinical therapeutic effectiveness (a), BMD (b, c), E2 (d), and TCM syndrome scores (e).

Eight RCTs reported a total effectiveness rate [24–26, 29–32, 34]. Three RCTs reported an effectiveness rate based on ACET alone [24–26]. However, the heterogeneity among the studies was substantial, with* I*^*2*^ = 78%,* P*=0.01. We identified the source of heterogeneity by performing sensitivity analysis; after removing one trial (Chen et al. 2010 [25]), we obtained a low heterogeneity, with* I*^*2*^ =38%,* P*=0.21. By contacting the author, the accuracy of the data in this paper was confirmed without publication bias. Therefore, the random effect model was adopted, but the subsequent analysis showed that there was no significant difference compared to the control group (RR= 1.11; 95% CI (0.89, 1.40);* P*=0.35). We consider it may be related to the small sample size (only three). Five RCTs reported an effectiveness rate of ACET combining calcium supplements [29–32, 34], and there was no heterogeneity among the trials (*I*^*2*^=0;* P=*0.0001). The results of the random effect model analysis showed a significant difference between ACET combining calcium supplements and the control group (RR= 1.32; 95% CI (1.20, 1.46);* P*<0.000 01) (Figure 3(a)). The publication bias (*P*=0.008) in Egger's test indicated that the publication bias was statistically significant. Subgroup analysis of the included studies was performed based on the different duration of treatment to compare the total effectiveness rate between the experimental and control groups. These results showed that there was high heterogeneity in studies using ACET alone for three months [25, 26] (*I*^*2*^=89%;* P=*0.003), and this high heterogeneity may have resulted from one trial (Chen et al. 2010 [25]). Random effect model of meta-analysis showed that there was no significant difference between treatment with ACET alone and the control group treatment for three-month studies (RR= 1.14; 95% CI (0.70, 1.85);* P=*0.59). However, subgroup analysis showed a significant difference between ACET combining calcium supplements and treatment with calcium supplements alone for six months [29–32, 34] (RR= 1.32; 95% CI (1.20, 1.46);* P*<0.000 01) (Table 2).

#### 3.4.2. BMD

Five RCTs reported the BMD [24, 26, 29, 31, 33]. Four RCTs reported the BMD of L2, L3, and L4 [26, 29, 31, 33]. The experimental groups of the three trials (Liu et al. [29], Liang et al. [31], and Lin et al. [33]) were treated with ACET combining calcium supplements, and the control group was treated with calcium supplements. However, the experimental group of the outlying trial (Wang et al. [26]) was treated with ACET alone, while the control group was treated with Western medicine. We obtained a low heterogeneity (*I*^*2*^=0%;* P=*0.96) through sensitivity analysis by removing Wang's trial. Therefore, the random-effect model was employed and the results showed a significant difference between the experimental and control groups (WMD= 0.03; 95% CI (0.01, 0.05);* P*=0.003), suggesting that ACET combining calcium supplements was more effective than calcium supplements alone for enhancing the BMD of L2, L3, and L4 (Figure 3(b)). Two RCTs reported the BMD of the femur-neck [24, 26]. We performed fixed-effect model analysis with no heterogeneity, and the results showed a significant difference between the ACET treatment and the use of medication alone on the BMD of the femur-neck (WMD= 0.07; 95% CI (0.03, 0.10);* P=*0.0006) (Figure 3(c)). The publication bias (*P*=0.042) in Egger's test indicated that the publication bias was statistically significant.

#### 3.4.3. E2

Eight RCTs reported the levels of E2 [23–27, 29–31]. Five RCTs of the included studies reported the E2 with ACET treatment alone [23–27], and the random effect model was adopted with high heterogeneity (*I*^*2*^=97%;* P*<0.000 01), and meta-analysis showed that there was no significant difference between the experimental and control groups (SMD= 0.27; 95% CI (-1.07, 1.60);* P=*0.70;* I*^*2*^=97%). We performed a sensitivity analysis by removing two trials (Gu et al. [24]; Ma et al. [27]) and obtained a result with low heterogeneity. The source of heterogeneity may be related to the different acupoints selected in the various studies. Nevertheless, there was no significant difference between the experimental and control groups (SMD= 0.20; 95% CI (-0.17, 0.57);* P=*0.28;* I*^*2*^=20%). Subgroup analysis showed that there was high heterogeneity in the intervention of ACET combining calcium supplements (*I*^*2*^=81%;* P=*0.005) [29–31]. The heterogeneity may result from the different units used to calculate the levels of E2 among the trials. [pg/mL] was used by Liu [29, 30], while [*μ*g/L] was employed by Liang [31]. Hence, a random effect model was adopted, and the results showed that ACET combining calcium supplements was more effective than calcium supplements alone based on the levels of E2 (SMD= 1.24; 95% CI (0.63, 1.84);* P*<0.0001) (Figure 3(d)). Egger's test reported that the publication bias* P*=0.082 indicated that there was no significant difference.

#### 3.4.4. TCM Syndrome Score

Three RCTs [28, 30, 31] reported the TCM syndrome score after three and six months of treatment. After three months of treatment, three trials showed a low heterogeneity (*I*^2^=19%;* P*=0.29), so a fixed-effect model was adopted. The meta-analysis appeared to show that ACET combining calcium supplements was more effective than calcium supplements alone in reducing TCM syndrome scores, with significant differences observed (WMD = -1.37; 95% CI (-1.76, -0.98); P<0.000 01). After six months of treatment, the included trials showed moderate heterogeneity (*I*^2^=45%;* P*=0.16). The meta-analysis results with a fixed-effect model were the same as those for three months (WMD= -2.37; 95% CI (-2.77, -1.97);* P*<0.000 01). The combined data showed that ACET combining calcium supplements was more effective than calcium supplements alone in reducing TCM syndrome scores, with significant differences (WMD = -1.85; 95% CI (-2.13, -1.57);* P*<0.000 01) (Figure 3(e)). The publication bias (*P*_three  months_=0.0195 and *P*_six  months_=0.113) in Egger's test indicated that there was no statistical difference.

#### 3.4.5. Patients' Quality of Life

**Figure 4:**
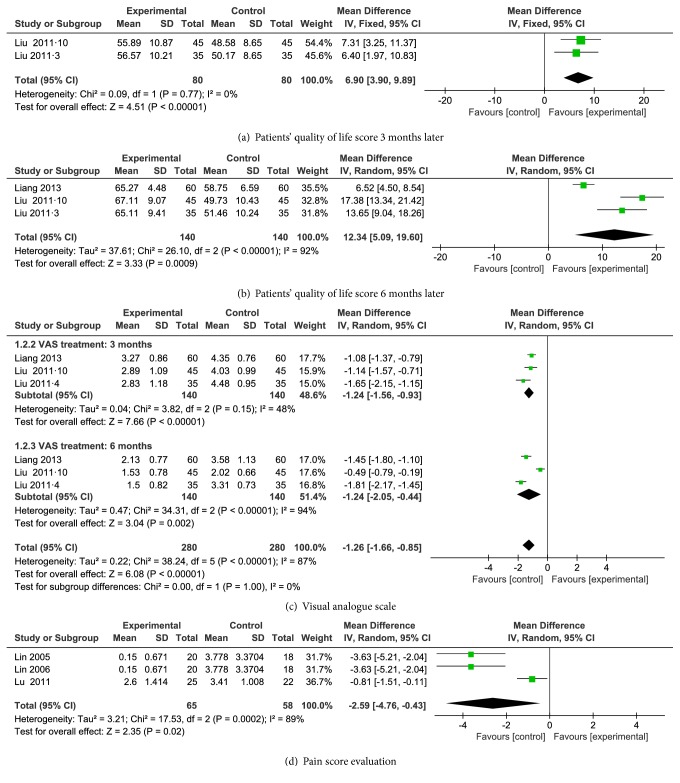
Meta-analysis of patients' quality of life (a, b), VAS (c), and pain score (d).

There were three RCTs reporting patient quality of life [28, 30, 31]. Two trials reported quality of life after three months of treatment [28, 30]. A fixed-effect model was adopted; there was no heterogeneity (*I*^2^=0;* P*=0.77), and the meta-analysis results appeared to show that, after three months of treatment, ACET combining calcium supplements was more effective than calcium supplements alone at improving the patient quality of life (WMD = 6.90; 95% CI (3.90, 9.89);* P*<0.00001) (Figure 4(a)). All three trials reported patient quality of life after six months of treatment. However, there was marked heterogeneity (*I*^2^=92%;* P*<0.000 01). We performed a sensitivity analysis and obtained a lower heterogeneity (*I*^2^=30%;* P*=0.23) after removing one trial (Liang et al. [31]). The high heterogeneity may have resulted from the differences in acupoint selection. Hence, a random effect model was adopted, and the meta-analysis appeared to show that, after six months of treatment, ACET combining calcium supplements was more effective than calcium supplements alone at improving the patient quality of life (WMD= 12.34; 95% CI (5.09, 19.60);* P*=0.0009) (Figure 4(b)). The publication bias (*P*_six  months_=0.244 ) in Egger's test indicated that there was no significant difference.

#### 3.4.6. Pain Assessment

Six RCTs reported pain assessment results based on a visual analogue scale (VAS) or pain score [29–34]. Three RCTs used a VAS (with a 10-point index: ranging from 0 to 10, the larger the number, the greater the degree of pain) to evaluate the degree of pain at the 3^rd^ and 6^th^ month after treatment [29–31]. There was medium heterogeneity (*I*^2^=48%;* P*=0.15) in the three-month treatment results and significant heterogeneity (*I*^2^=94%;* P*<0.000 01) at six months. The high heterogeneity may be related to differences in acupoint selection. Therefore, a random effect model was adopted, and the meta-analysis results showed that ACET was more effective than calcium supplements alone in relieving pain (WMD = -1.26; 95% CI (-1.66, -0.85);* P*<0.000 01). Further subgroup analysis showed that, after both three and six months of treatment, ACET combining calcium supplements was more effective than calcium supplements alone in reducing the VAS scores (WMD after three months = -1.24; 95% CI (-1.56, -0.93);* P*<0.000 01; and WMD after six months = -1.24; 95% CI (-2.05, -0.44);* P*=0.002) (Figure 4(c)).

The other three trials used a pain score, which includes the evaluation of resting pain (i.e., the pain occurring at rest) and movement-evoked pain (i.e., the pain presenting during activity) to assess the degree of pain after six months of treatment [32–34]. As a result of high heterogeneity, sensitivity analysis was adopted, and we obtained a lower heterogeneity by removing one trial (Lu et al. [34]). The high heterogeneity may result from differences in the calcium supplements used. We used the random effect model, and the analysis results showed that ACET combining calcium supplements was more effective than calcium supplements alone in reducing pain scores (WMD= -2.59; 95% CI (-4.76, -0.43);* P*=0.02) (Figure 4(d)).

### 3.5. Adverse Reaction

Six of the twelve RCTs reported adverse reactions to the interventions [23, 25, 28–31]. Two trials only descriptively reported that there was no infection, fainting during acupuncture, haematoma formation, or foreign-body reaction in either the experimental or control group [24, 25]. Four trials specifically reported the number of participants and the symptoms and solutions of adverse reactions [23, 28–31]. The main adverse reactions of the experimental group included fainting, haematoma formation, and foreign-body reactions, among others; the control group reactions included nausea, drowsiness, and breast distention, among others. The incidence of adverse reactions was 12.6% (28/223) in the experimental groups compared to 8.1% (18/222) in the control groups. The adverse reactions of the two groups were mild to moderate in severity and easy to tolerate.

### 3.6. Frequency of Selected Acupuncture Points

**Figure 5:**
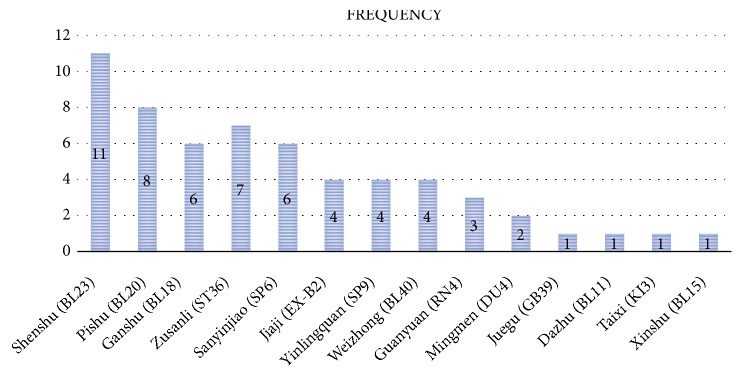
The most frequently used acupoints in these trials.

Microsoft Excel 2016 was used to calculate the frequency of the use of selected acupuncture points, and we found that Shenshu (BL23), Pishu (BL20), Zusanli (ST36), Ganshu (BL18), and Sanyinjiao (SP6) were the five points used most frequently (Figure 5).

### 3.7. Trial Sequential Analysis

**Figure 6:**
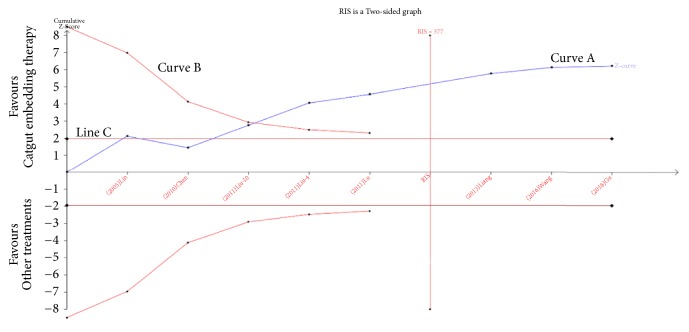
Trial sequential analysis for ACET effectiveness in the treatment of PMOP.

Our TSA analysis depicted that cumulative Z curve crossed the trial monitoring boundary after the inclusion of the fourth trial. The dominant model was taken as an example in the TSA analysis, which indicated that ACET can increase the total effectiveness of PMOP (RR=1.25) and hence no further trials are required (Figure 6).

## 4. Discussions

The objective of the meta-analyses was to evaluate the effectiveness and safety of ACET for the treatment of PMOP. The combined data indicated that ACET alone or ACET in combination with calcium supplementation was superior to the interventions in the various control groups in terms of levels of BMD, pain relief, patients' quality of life, and TCM syndrome score, among others. However, the effects of ACET alone on the effectiveness rate were similar to those observed with medication alone, while ACET combining medicine supplementation was superior on it. As we can see from the forest plot, only Chen [25] declared that there was no statistical difference between the experimental and control group. According to Table 1 (Characteristics of the Included Studies), we found that the control group of Chen's trial [25] was treated with Fufuchun capsule (estrogenic medicine) while the other two control groups were treated with calcium supplements. It is well known that the effectiveness of estrogen for PMOP is recognized internationally. In addition, Chen [25] adopted Kuppermann to evaluate the effectiveness, including four levels: absolutely clinical relief, obvious improvements, some improvements, and no improvement. In meta-analysis, the effective number was defined as the total number minus the invalid number, and there was no grading score. However, absolutely clinical relief and obvious improvement are still the ideal therapeutic effects in clinical treatment. Meanwhile, effectiveness rate is a subjective indicator, and most patients still believe that the treatment of PMOP is medicine-based. As a new treatment, ACET has not been approved by the majority and there may be expectation bias on the result [35]. To sum up, the effectiveness of ACET alone for the treatment of PMOP remains to be further researched, and we are optimistic in this respect.

It is well known that pain relief is the greatest feature of acupuncture therapy. Four RCTs suggested that the pain relief effect of a number 9 syringe needle (the primary tool for ACET) in painful areas was similar to that of dry needling [28–31], which relieves pain by releasing muscle adhesions [36]. A large number of studies have indicated that ACET has analgesic effects, which may be related to the regulatory effects of ACET on phenylalanine, glycine, and endocannabinoid levels [37].

The major cause of PMOP is the reduction of oestrogen secretion in postmenopausal women whose hypothalamic-pituitary-ovarian axis function is in decline. The activity of osteoclasts cannot be inhibited by low levels of oestrogen, leading to imbalances in bone resorption and bone formation. In addition, the abnormal differentiation of bone marrow mesenchymal stem cells (BMMSCs) can also cause this disease [38]. ACET has similar oestrogen-like effects and can regulate bone mass extensively through the three hypothalamic-pituitary axes: the hypothalamus-pituitary-ovarian axis, the hypothalamus-pituitary-adrenal axis, and the hypothalamus-pituitary-thyroid axis [39, 40].

The acupoints used in the twelve included RCTs were different; however, five points [Shenshu (BL23), Pishu (BL20), Zusanli (ST36), Ganshu (BL18), and Sanyinjiao (SP6)] were most commonly used. In TCM theory, PMOP results from an insufficiency of kidney essence and liver-kidney depletion. The treatment principle should tonify the liver and kidney to strengthen bone and tendon. Therefore, the major acupoints are related to the three meridians of the liver, spleen, and kidney. Shenshu (BL23) and Ganshu (BL18) are the Back-shu acupoints, which the Qi of viscera and bowels transport and infuse into, and they can treat disease of the corresponding viscera and bowels. Sanyinjiao (SP6) is the point at which the three meridians of the liver, spleen, and kidney connect and can adjust the three meridians simultaneously. Pishu (BL20) and Zusanli (ST36) can tonify the spleen and stomach, the root of acquired constitution. Hence, the combination of these acupoints could tonify the roots of innate endowment and acquired constitution together.

The results of this systematic review show that ACET appears to offer certain benefits to PMOP patients. However, this review has the following limitations: (1) Only twelve RCTs were included in this meta-analysis and the methodological quality of existing clinical RCTs was generally low due to lack of the description of randomized method, blinding method, allocation and concealment methods, and reports of the loss to follow-up or withdrawal. An intention-to-treat (ITT) analysis was also not performed. (2) The large heterogeneity among the studies may be related to the different intervention measures, acupoint selection, and even the skill level of the acupuncturists, which is a common problem in acupuncture and moxibustion therapy. (3) There were subjective indicators included in the outcomes, which may be influenced by memory bias or measurement bias. And the effectiveness rate is not an internationally recognized outcome indicator. (4) Compared with medicinal therapy, ACET cannot be blinded; this is the biggest problems of nonmedicinal treatment. The design of sham ACET can guarantee the implementation of the blinded, but it involved certain ethical issues. The overall results of the trials may have been affected, and some biases in the meta-analysis conclusions were unavoidable.

In summary, ACET could offer some clinical benefits to PMOP patients. However, the overall reliability of this conclusion was somewhat lessened because of the limitations in the quality and quantity of the included RCTs. It is suggested that researchers should strive to perfect their clinical trial designs as much as possible. Studies should consistently report randomization methods, allocation concealment protocols, and patient drop-out or withdrawal information in detail, while subjective outcome indicators should be avoided. Future clinical researchers should perform multicentre, large-scale RCTs in the future. Acknowledged and unified curative criteria as outcomes should be adopted to more definitively evaluate the effectiveness and safety of ACET.

## Figures and Tables

**Table 1:** Characteristics of the included studies.

Included studies	Participants	Intervention		Duration (months)	Age	Outcome	Jadad Score
	(Experimental group/Control group)	Experimental group	Control group				
Zhou 2014 [23]	15/15	ACET (BL23+BL20+ST36)	Sham ACET	3	58±7.05	(3) (4) (11) (12)	4

Gu 2018 [24]	30/30	Stellate ganglion embedding: 2-3 cm above the sternoclavicular joint and 1.5 cm from the median line	Sodium alendronate	6	62±5	(1) (3) (4)	2

Chen 2010 [25]	33/32	ACET (BL23+SP6+RN4) and (BL18+ST36+BL20) would be used according to the TCM syndrome differentiation	Fufuchun capsule	3	53.85±2.45	(1) (3) (4) (5) (6)	3

Wang 2016 [26]	30/30	ACET (BL20+BL23+ST36+GB39+ BL11+KI3)	Nylestriol	3	62.22±26.01	(1) (2) (3) (4) (7)	2

Ma 2010 [27]	96/96	ACET (BL23+DU4+RN4) and (BL15+BL18+BL20+SP6) as adjunct	Nylestriol+ Oryzanol	3	55.3	(3) (4) (8) (9) (10) (13)	1

Liu 2011 [28]	35/35	Calcium supplements + ACET (BL23+BL18+EX-B2+BL40) and (SP6+BL20+ST36+SP9) would be used according to the TCM syndrome differentiation	Calcium supplements	6	62.2±2.8	(1)	3

Liu 2011 [29]	35/35	Calcium supplements + ACET (BL23+BL18+EX-B2+BL40) and (SP6+BL20+ST36+SP9) would be used according to the TCM syndrome differentiation	Calcium supplements	6	62.7±6	(1) (2) (3) (4)	3

Liu 2011 [30]	45/45	Calcium supplements +ACET (BL23+BL18+EX-B2+BL40) and (SP6+BL20+ST36+SP9) would be used according to the TCM syndrome differentiation	Calcium supplements	6	61.8±8.4	(1) (2) (3) (4)	3

Liang 2013 [31]	60/60	Calcium supplements + ACET (BL23+BL18+EX-B2+BL40) and (SP6+BL20+ST36+SP9) would be used according to the TCM syndrome differentiation	Calcium supplements	6	61.8±8.4	(1) (2) (3) (4)	2

Lin 2005 [32]	20/18	Leli capsules + ACET (BL23)	Leli capsules	6	60.06	(1) (2)	2

Lin 2006 [33]	20/18	Leli capsules + ACET (BL23)	Leli capsules	6	61.2	(2) (4)	2

Lu 2011 [34]	25/22	Calcium supplements + ACET (BL23)	Calcium supplements	6	61.49	(1) (2) (3) (4)	2

Notes: (1) total effectiveness rate; (2) pain visual analogue scale (VAS); (3) level of E2; (4) BMD; (5) bone metabolism; (6) hepatic and renal function; (7) levels of BGP and CT; (8) level of LH; (9) level of FSH; (10) levels of serum calcium and phosphorus, ACP, and urinary DPD/Cr; (11) urinary calcium/chromium; (12) level of interleukin 6 (IL-6); (13) changes in the endometrium.

**Table 2:** Subgroup analysis of total effectiveness rates with different treatment duration.

Duration	Subgroup analysis result		*P*	*I* ^*2*^
	Number of trials	RR (95% CI)		
Three months	2[25, 26]	1.14 (0.70, 1.85)	0.59	89
Six months	5[29-32, 34]	1.32 (1.20, 1.46)	<0.00001	0

## Supplementary Materials

Supplementary Materials PRISMA 2009 checklist: the effectiveness and safety of acupoint catgut embedding for the treatment of postmenopausal osteoporosis: a systematic review and meta-analysis.
